# Determinant of time-to-first birth interval after marriage among Ethiopian women

**DOI:** 10.1186/s12905-019-0858-3

**Published:** 2019-12-10

**Authors:** Ayele Gebeyehu Chernet, Dinberu Seyoum Shebeshi, Akalu Banbeta

**Affiliations:** 1 Department of Statistics, College of Natural and Computational Sciences, Wolkite University, Wolkite, Ethiopia; 2 Department of Statistics, College of Natural and Computational Sciences, Jimma University, Jimma, Ethiopia

**Keywords:** Frailty, First birth interval, Acceleration factor, Ethiopia

## Abstract

**Background:**

Time-to-first birth after marriage has a significant role in the future life of each individual woman and has a direct relationship with fertility. This study aimed to see the determinant of time-to-first birth interval after marriage among Ethiopian women.

**Methods:**

The data was obtained from 2011 Ethiopia Demographic and Health Survey which is the third survey. The sample was selected using a stratified; two-stage cluster sampling design and the data was analysed using parametric shared frailty model.

**Results:**

A total of 7925 ever married women from the nine region of the country were included in this study. Of the total women, 5966 (75.3%) of them gave firstbirth. Age, residence area, employment status, contraceptive use and education of women were associated significantly to time-to-first birth.

**Conclusions:**

Women having younger age at first marriage, urban women, contraceptive users had prolonged time to first birth interval. There is a need of teaching family for contraceptive use and improving women education to increase the length of first birth interval in Ethiopia.

## Background

The first visible outcome of the fertility process is the birth of the first child. The first birth marks a woman’s transition into motherhood. It plays a significant role in the future life of each individual woman and has a direct relationship with fertility [1]. The timing of the first birth influences the number of children a woman bears throughout her reproductive period in the absence of any active fertility control, and woman who starts giving the first birth very early in life tends to have a large number of children than those who starts late [2].

Fertility patterns in the world have changed dramatically over the last two decades since the international conference on population and development (ICPD) in 1994, producing a world with very diverse child bearing patterns [3]. Many countries in Asia were able to reduce their fertility through government policies. For instance, China and Vietnam have witnessed declines in their total fertility rate (TFR) due to stringent government policies that discourage early and arranged marriage [4]. However, the delayed fertility transition has been observed to be underway in the region with remarkable progress in African countries like South Africa, Botswana, and Zimbabwe [5], fertility remains high in Africa by the standards of the rest of the world.

Fertility rates in sub Saharan Africa have been identified to exhibit a very unique demographic scenario in the world that sets it apart from other regions in the world. Contrary to the case of most regions like Europe, South America and Asia that have for long entered the fertility transition marked by a declined in their fertility rates in the 1950s and 1960s, sub-Saharan Africa is the only region in the world, where fertility decline has been rather slow and late [6]. Fertility rates in the sub-continent stand at the same level as that of Asia and South America towards the end of the 1970s. Most countries in Sub Saharan Africa are still experiencing relatively higher fertility rates. What can be discern from the information so far provided, is that sub-Saharan Africa is the sole region in the world that has not so far experienced any significant decline in its fertility rates [7]. According to United Nations 2014 report, 45 out of 66 high fertility countries (more than 3.2 children per woman) are increasingly concentrated in sub-Saharan Africa [3].

Ethiopia is the second most populous countries in sub-Saharan Africa next to Nigeria with 94,351,001 population size and 29 years of doubling time along with the scarcity of resources [8]. Uncontrolled fertility has adversely influenced the socio-economic, demographic and environmental development of the country. Poverty, war and famine, associated with low levels of education and health, a weak infrastructure, and low agricultural and industrial production have aggravated the problem of overpopulation [9]. When we look back at the history of Ethiopia population growth rate, there has been a steady increase since 1960. Based on 1984 census information, population growth rate was estimated at about 2.3% for the 1960–70 period, 2.5% for the 1970–80 period, and 2.8% for the 1980–85 period. Population projections compiled in 1988 by the CSA projected a 2.83% growth rate for 1985–90 and a 2.96%t growth rate for 1990–95. According to the 2007 Ethiopia population census, the annual population growth rate within 1994–2007 was estimated as 2.6%.

Age at the start of marriage is one of determinant factors. Early childbearing can interrupt a young women’s education and other activities which women need to accomplish [3]. Other major social, economic and cultural factors which are related to family and reproduction status, as well as personal values and practices, are shown to influence fertility and thus time to first birth [4, 5].

But the fertility rate is quite different across different customs, culture and practice of people living in different regions of Ethiopia. This implied that the existence of heterogeneity in the survival of time-to-first birth between different regions. For the formulation of effective policy to motivate people for longer first birth interval after marriage, it is crucial to study the effect of various socio-economic and demographic factors which affect time-to-first birth. Having these, this research aimed to examine factors associated to time-to-first birth interval after marriage using parametric shared frailty survival models.

## Methods

### Study population and sampling design

The data was obtained from 2011 EDHS, which was taken from Central Statistical Agency (CSA). Each region of Ethiopia was stratified into urban and rural areas resulting 21 sampling strata. Enumeration Areas (EAs) were selected independently in each stratum in two stages. In the first stage, a total of 645 EAs (202 EAs in urban areas and 443 EAs in rural areas) were selected with probability proportional to the EA size (based on the 2007 Population and House Census) and with independent selection in each sampling stratum. The resulting lists of households served as a sampling frame for the selection of households in the second stage. In the second stage of selection, a fixed number of 28 households per cluster were selected with an equal probability systematic selection from the newly created household listing.

The questionnaires for data collection were adapted from model survey instruments developed for the measure DHS project to reflect the population and health issues relevant to Ethiopia. The questionnaires were developed in English and translated into three major local languages-Amharic, Oromiffa, and Tigrigna. A representative sample of 17,817 households were selected, of these, 16,702 were successfully interviewed. In the interviewed households 17,385 eligible women were identified for individual interview; complete interviews were conducted for 16,515. After excluding missing values, a total of 7925 with women were included [10].

### Variables and statistical analysis

#### Response variable

The response variable is time-to-first birth among woman in Ethiopia, which is measured in months. For women who did not give birth (censored) the time was measured till the date of the interview. Independent Variables: Independent variables included in the analysis are described in Table 1 as follows.

**Table 1:** Description of independent variables used in the analysis

Variables	Description	Categories
age	Age of women at marriage	Measured in years
Women education	Women’s level of education	0 = No education;1 = Primary; 2 = Secondary &above
Husband education	Husband’s level of education	0 = No education;1 = Primary; 2 = Secondary and above
Wealth index	Household wealth index	0 = Poor; 1 = Meddle; 2 = Rich)
Place of residence	Place of residence	0 = Rural; 1 = Urban)
Mass media	Access to mass media	0 = No; 1 = Yes
Employment	Employment status	0 = unemployed,1 = Employed
Contraceptive	Use of Contraceptive	0 = Non-User, 1 = User
Religion	Religion of respondents	0 = Muslim,1 = Orthodox,2 = Protestant, 3 = Other
Region	Region of respondents	Tigray, Afar, Amhara, Oromia, Somali, Benishangul, SNNPR, Gambela, Harari, Addis Ababa, Dire Dawa

Region of the women was considered as a clustering effect in frailty model

### Statistical analysis

Descriptive analysis was performed using frequency and percentage for both dependent and independent variables. Parametric Shared Frailty survival model was used to identify significant factors of FBI by considering region of women as clustering variable because fertility rate is different due to the presence of different custom; culture and practice of people across regions of the country. The shared frailty approach assumes that all FBI in a cluster are conditionally independent given the frailties. The value of the frailty term is constant over time and common to all individuals in the cluster, and thus it is responsible for creating dependence between event times in a cluster [11, 12].

Akaike’s information criterion (AIC) was used for model comparison. Data cleaning, management and analysis were carried out using STATA, Version 12. All hypotheses testing to determine differences, associations and relationships were judged significant at *p* < 0.05.

### Inclusion exclusion criteria

Women who went into marriage for the first time without a child or no pregnancy and with complete records were considered. Thus, women having less than nine months of waiting time for first birth after marriage and having negative birth interval were excluded.

### Limitation of study

This research excluded Somali region because the data cannot be considered as representatives of the region since some EAs are not interviewed due to drought and security problems. And also some factors which may have significant contribution to FBI such as age at menarche and menstrual status were not considered. Investigating the influence of these factors may be appropriate to understand further dynamics of time to first birth after marriage in the country. In addition to this, further studies should be warranted in each region of Ethiopia and explore other factors that are not addressed in this study.

## Results

A total of 7925 women who got first marriage from eight regional states and two administrative cities of Ethiopia were included. Of the total women, 5966 (75.3%) of them gave first birth while 1959 (24.7%) of them did not give birth until the end of the interview (Table 2). The median time of FBI and age of women at first marriage were estimated to be 30 months and 16 years respectively.

**Table 2:** Baseline covariates characteristics with their time-to-event status

	Categories	Total (%)	Status	
Variable			Censored (%)	Event (%)
Place of Residence	Rural	5969 (75.3)	1155 (19.4)	4814 (80.6)
	Urban	1956 (24.7)	804 (41.1)	1152 (58.9)
Wealth Index of	Poor	3310 (41.8)	608 (18.4)	2702 (81.6)
Family	Middle	1260 (15.9)	244 (19.4)	1016 (80.6)
	Rich	3355 (42.3)	1107 (33)	2248 (67)
Contraceptive Use	Use	5901 (74.5)	1514 (25.7)	4387 (74.3)
	Not Use	2024 (25.5)	445 (22)	1579 (78%)
Employment status of	Yes	2935 (37.0)	966 (32.9)	1969 (67.1)
Women	No	4990 (63)	993 (19.9)	3997 (80.1)
Religion	Muslim	2925 (36.9)	579 (19.8)	2346 (80.2)
	Orthodox	3220 (40.6)	1017 (31.6)	2203 (64.4)
	Protestant	1535 (19.4)	312 (20.3)	1223 (70.7)
	Other	245 (3.1)	51 (20.8)	194 (79.2)
Mass Media	No	4585 (57.9)	986 (21.5)	3599 (78.5)
	Yes	3340 (42.1)	973 (29.1)	2367 (70.9)
Women’s’ Education	No education	5108 (64.4)	1169 (22.9)	3939 (77.1)
Level	Primary	2159 (27.2)	507 (23.5)	1652 (76.5)
	Secondary & above	658 (8.3)	283 (43)	375 (57)
Husband’s Education	No education	3857 (48.7)	939 (24.3)	2918 (75.6)
level	Primary	2855 (36.0)	569 (19.9)	2286 (80.1)
	Secondary& above	1213 (15.3)	451 (37.2)	762 (62.8)
Total			1959 (24.7)	5966 (75.3)

The median survival time to first birth with the corresponding 95% confidence interval for all region of the country was presented in Table 3. The maximum and minimum median time to first birth for women was from Amhara (51 months) and SNNP (23 months) region respectively.

**Table 3:** Median survival time (in months) of first birth by region

Region	Number of women (%)	Median (IQR)
Addis Ababa	548 (6.9)	37 (50)
Affar	718 (9.1)	26 (31)
Amhara	1166 (14.7)	51 (61)
Beninshangul- Gumuz	684 (8.6)	33 (51)
Dire-Dawa	516 (6.5)	30 (41)
Gambela	636 (8.0)	31 (41)
Harari	543 (6.9)	28 (51)
Oromiya	1203 (15.2)	24 (26)
SNNP	1092 (13.8)	23 (25)
Tigray	819 (10.3)	37 (46)

*IQR* Inter Quartile Range

The result of log-normal gamma shared frailty model was presented in Table 4. The age of women at marriage was statistically significant to determine time-to-first birth after marriage, the acceleration factor (ϕ = 0.927, 95% CI: 0.922–0.932). Accordingly, as age of women at first marriage increases, the time-to-first birth becomes shorter by a factor of ϕ =0.927.

**Table 4:** Results of log-normal gamma shared frailty model

Covariate	Category	Estimate($\hat{\beta}$)	SE($\hat{\beta}$)	ϕ	95% CI	*P*-value
Age		−0.08	0.0027	0.927	[0.922, 0.932]	0.000*
Place of	Rural	Ref.				
Residence	Urban	0.26	0.0250	1.292	[1.231, 1.357]	0.000*
Employment	Unemployed	Ref.				
Status	Employed	0. 08	0.0180	1.080	[1.042, 1.120]	0.000*
Contraceptive	Non- User	Ref.				
	User	0.11	0.0206	1.116	[1.072, 1.162]	0.000*
Women	No education	Ref				
Education	Primary	−0.19	0.0204	0.828	[0.796, 0.862]	0.000*
	Sec & above	−0.05	0.0395	0.956	[0.885, 1.033]	0.259
*θ* = 0.78	*λ* = 2.541				likelihood = − 813.8	
*τ* = 0.28	*ρ* = 3.185				AIC = 13,641.69	
Likelihood-ratio test of *θ* = 0: chi-square = 1307.52 P-value = 0.000*						

ϕ Indicates Acceleration factor;* significant at 5% level; 95%CI: 95% confidence interval for acceleration factor; SE($\hat{\upbeta}$): standard error for $\hat{\upbeta}$; Ref. Reference

Urban women have prolonged time-to-first birth than women from rural by a factor of ϕ =1.292. Employed women prolonged time-to-first birth than unemployed women by a factor of ϕ = 1.080 with 95% CI: 1.042–1.120. Mothers who used contraceptive methods prolonged time-to-first birth by a factor of ϕ = 1.116 (95% CI: 1.072–1.162) as compared to non-users. Women who attended secondary education and above had shorten time-to-first birth by a factor of ϕ = 0.828 with 95% confidence interval (0.796, 0.862) as compared to the uneducated women. The variance of the frailty distribution (**θ** = 0.78) had significant contribution to the model that indicates the presence of heterogeneity among timing of first birth among the regions.

## Discussion

This study applied parametric survival frailty models in order to investigate the determinant factors of time-to-first birth after marriage among Ethiopian women, based EDHS 2011 data set. 7925 Ethiopian women who got an official marriage were included in the study. The prevalence of having the first child after marriage was 75.3%. Graphically it could be observed that high rate of first birth within the first three years following the marriage. This finding similar to the previous community based cross-sectional study in Lemo district, Ethiopia [1]. Previous study in Sub-Saharan level was established that 57% of women of child bearing age in 2002 practiced time to first birth less than 3 years [6]. This may be due to the reason that many women in developing countries do not use contraception after birth and therefore are likely to become pregnant once fecundity achieved [13].

The estimated median age of women at marriage was 16 years. This result is consistent withstudy conducted in the county where 60.6% of Ethiopian women were married before the ageof 18 years [14].

The estimated median survival time of first birth after marriage of Ethiopian women is found to be 30 months. This finding is almost similar with the finding for the same country using 2005 EDHS [15] and result revealed that the median time of FBI was 29 months. This estimate is also exactly identical to Ghanaian women [16]. However, the median time of FBI is a bit lower than women in Bangladesh where it was estimated to be 25 months [17]. This difference may be due to the practice of early marriage in Ethiopia which had potential to prolong timing of first birth [15].

Marriage at older age significantly associated with shorter time interval for the first birth. This result is consistent with the study in the country and abroad [18, 19]. The reason may be older women need to give birth soon after marriage to have the desired number of children before the end of their reproductive life. And, woman who gets early marriage use contraceptive to elongate time-to-first birth until it becomes physically mentally matured [17]. However, some contradictory results were also observed such as in Pakistan, younger women at marriage had shorter FBI [20].

The results of this study suggested that women who lived in urban areas had longer first birth interval than women who lived in rural areas. Rural inhabitants have usually no access for maternal health and family planning programs as compared to urban residents [15] which may results in short birth interval. This finding is supported by a study in Bangladeshi study and a longitudinal data based study in Nigeria [16, 17]. Another important finding of this study was that employment status of the women had a significant association to time-to-first birth. Time-to-first birth interval following marriage for employed women was longer than unemployed women. This is consistent with studies which were done abroad [21, 22]. Women those used contraceptive had long time-to-first birth than the non-users. This is due to contraceptive service which helped them to protect early and unwanted pregnancy in marriage life of the couples [17]. A similar study in India reported that contraceptive use did not emerge as a significant associated factor at any birth interval [23].

A significant association was found between women education and time-to-first birth. Women with secondary and above education have significant shorter first birth interval as compared to women who were not educated. This may be due to the reason that at the time of entry to marriage life, educated women can be emotionally prepared, biologically matured, and financially secured to have a child. A study in India established that education of women was significantly associated with first birth interval only while husband’s education was significantly associated with first and second birth interval [23]. In another study on time-to-age at first birth in Ethiopia, they established that educated women have longer waiting time to time-to-first birth after marriage. When women spend a longer time at school, this is likely to significantly affect both age at marriage and the duration between marriage and the first birth [24].

## Conclusions

The study aimed to examine time-to-first birth interval after marriage and its associated factors among Ethiopian women using Parametric Shared Frailty Model. A total of 7925 woman who got involved in first marriage from the nine region of the country included in this study. The median survival time of FBI was estimated to be 30 months. Younger age, residence area, employment status, contraceptive use and education status of woman associated significantly to time to FBI. There is a need of teaching family for contraceptive use and improving woman education to increase the length of FBI along with not encouraging early marriage to get control a rapid population growth in Ethiopia.

## Data Availability

The dataset was demanded and retrieved from CSA website after formal online registration and submission of the project title and description. The data can be accessed through http://www.statsethiopia.gov.et/.

## Abbreviations

ANC: Antenatal Care
CSA: Central Statistical Agency
EAs: Enumeration Areas
EDHS: Ethiopian Demographic and Health Survey
FBI: First Birth Interval
HH: Household
SE: Standard Error
WHO: World Health Organization
